# Diet and Host Genetics Drive the Bacterial and Fungal Intestinal Metatranscriptome of Gilthead Sea Bream

**DOI:** 10.3389/fmicb.2022.883738

**Published:** 2022-05-06

**Authors:** Fernando Naya-Català, M. Carla Piazzon, Josep A. Calduch-Giner, Ariadna Sitjà-Bobadilla, Jaume Pérez-Sánchez

**Affiliations:** ^1^Nutrigenomics and Fish Growth Endocrinology Group, Institute of Aquaculture Torre de la Sal Spanish National Research Council (IATS-CSIC), Valencia, Spain; ^2^Fish Pathology Group, Institute of Aquaculture Torre de la Sal Spanish National Research Council (IATS-CSIC), Valencia, Spain

**Keywords:** metatranscriptome, teleost fish, bacteria, fungi, archaea, virus, selective breeding, nutrition

## Abstract

The gut microbiota is now recognised as a key target for improving aquaculture profit and sustainability, but we still lack insights into the activity of microbes in fish mucosal surfaces. In the present study, a metatranscriptomic approach was used to reveal the expression of gut microbial genes in the farmed gilthead sea bream. Archaeal and viral transcripts were a minority but, interestingly and contrary to rRNA amplicon-based studies, fungal transcripts were as abundant as bacterial ones, and increased in fish fed a plant-enriched diet. This dietary intervention also drove a differential metatranscriptome in fish selected for fast and slow growth. Such differential response reinforced the results of previously inferred metabolic pathways, enlarging, at the same time, the catalogue of microbial functions in the intestine. Accordingly, vitamin and amino acid metabolism, and rhythmic and symbiotic processes were mostly shaped by bacteria, whereas fungi were more specifically configuring the host immune, digestive, or endocrine processes.

## Introduction

The green transition of food production systems is the main issue to address the increase of production volumes. Thus, the target is to decrease the environmental footprint, while complying with high food safety and quality standards (Béné et al., 2015; FAO, 2016). However, climate change will impact most food production systems, and aquaculture, in particular, both directly affecting the physical condition and physiology of stocked animals, and indirectly altering fishmeal and fish oil costs, as well as other goods and services (Bohnes et al., 2019; Garrett et al., 2021). To overcome some of these constraints, continuous efforts have been conducted over the last two decades for the successful replacement of marine feed ingredients with more sustainable feedstuffs in fish feeds (Benedito-Palos et al., 2007; Lazzarotto et al., 2018; Perera et al., 2019; Egerton et al., 2020). The most obvious alternatives are plant proteins and oils, and, interestingly, most drawbacks effects - including pro-inflammatory condition, loss of epithelium integrity, and advanced male-female sex reversal in a protandrous hermaphrodite fish such as gilthead seabream (*Sparus aurata*), - can be restored, at least partially, with the use of feed additives (Estensoro et al., 2016; Piazzon et al., 2017; Simó-Mirabet et al., 2018; Reis et al., 2021). In addition, a range of novel feed formulations, including emerging feed ingredients (i.e., insects, seaweeds, microalgae, microbial biomasses, land-animal processed animal proteins, fish meal, and oil from fisheries and aquaculture by-products), do not affect fish welfare and guarantee good zootechnical performance in gilthead sea bream (Basto et al., 2021; Naya-Català et al., 2021a; Solé-Jiménez et al., 2021). Therefore, it is possible to produce marine seafood products using formulation concepts and ingredients that fit into a circular economy framework.

Genetic improvements in feed conversion ratio (FCR) also contribute to moving more toward a more environmentally-sustainable aquaculture sector (Kause et al., 2016; Vandeputte et al., 2019), though FCR is a problematic trait to be included in aquaculture breeding programmes due to the difficulties of accurate measurements of individual feed intake in the aquatic milieu (De Verdal et al., 2018; Besson et al., 2019). Thus, in gilthead sea bream, most genetic selection programmes have been applied for the direct selection and improvement of somatic growth, disease resistance, or carcass quality (Perera et al., 2019, 2021b; Lorenzo-Felipe et al., 2021). However, there is now evidence that selection for growth in a farming environment co-selects changes in reproductive or swimming performance (Ferosekhan et al., 2021; Perera et al., 2021a). In addition, we have recently reported that selection for growth also selects for a more functionally flexible microbiota when the inferred gut metagenomes of representative fish families with different growth trajectories across the production cycle were compared (Piazzon et al., 2020). However, such an approach was based on the amplification of specific variable regions (v3-v4) of the 16S rRNA gene, and they only inform about the taxonomic profile of one portion (Bacteria, Archaea) of the whole gut microbial community, which also includes Fungi and Virus (Merrifield and Rodiles, 2015). In addition, the inferred functional changes related to gut bacteria variations might not correlate with the actual expression profile of these populations. To solve these issues, metatranscriptomic analyses are perhaps a better approach (Aguiar-Pulido et al., 2016), evidenced by the exponential increase of metatranscriptomic projects over the last 20 years (Shakya et al., 2019). Such approach has been used in humans to characterise active microbes in a community, discover novel microbial interactions, and track the relationship between viral genes and their hosts (Gosalbes et al., 2011; Bikel et al., 2015; Bashiardes et al., 2016; Moniruzzaman et al., 2017). In livestock species, metatranscriptomics has helped to reveal the association between breed effect and rumen microbiome activity (Li et al., 2019a). In aquatic organisms, although at a lower extent, there are also some examples analysing the composition of marine fish viromes (Geoghegan et al, 2021), to characterize the full set of water-living microbes (Salazar et al., 2019; Trench-Fiol and Fink, 2020), and to reveal microbial functions associated with the digestion of algal polysaccharides in the digestive tract of the abalone *Haliotis discus hannai* (Nam et al., 2018). Yet, to date, there is no information on the genome × gut metatranscriptome interaction of genetically selected fish and how this can affect their nutritional plasticity. This is, thereby, the aim of the present study, where we sequenced the intestinal metatranscriptome of two gilthead sea bream families with opposite growth trajectories from the study of Piazzon et al. (2020) with three main objectives: i) to study and characterize the full set of microbes present in the gut of this fish species, ii) to evaluate if genetic background and alternative diets can change the expression of genes collectively expressed by these microbial communities, and iii) to unravel which metabolic processes are enriched in gilthead sea bream intestine and which microbial community is involved, to provide more insights into the broad microbial landscape of the gut of farmed fish.

## Results

### Samples

**FIGURE 1:**
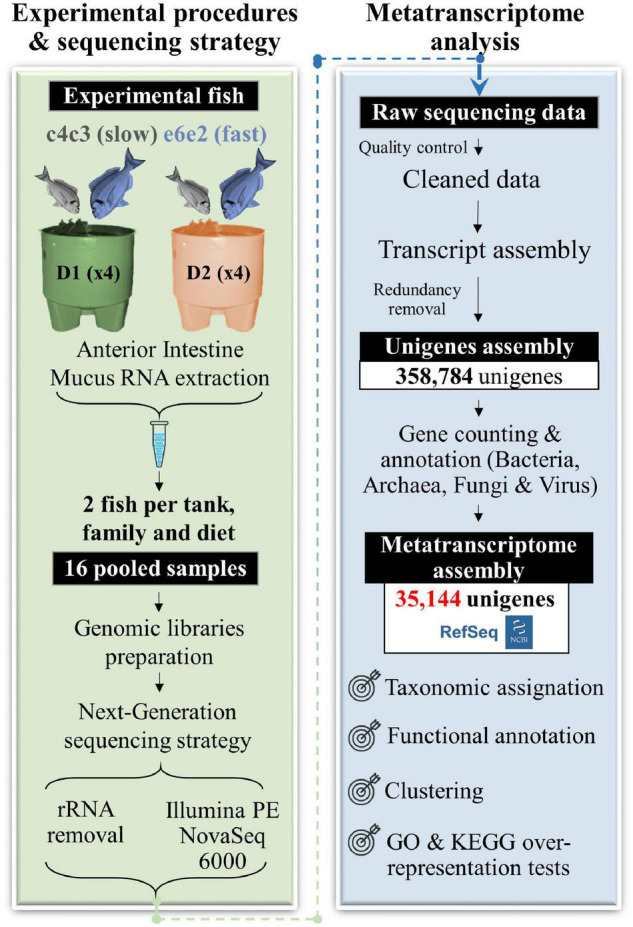
Workflow of the gilthead sea bream metatranscriptome assembly conducted in this study. Black boxes with white text indicate generated genomic resources, according to the following steps: experimental procedures and sequencing, metatranscriptome assembly, and post-assembly analyses (taxonomic assignation, functional annotation, and GO and KEGG over-representation tests).

Two gilthead families from the PROGENSA^®^ breeding programme (Perera et al., 2019, 2021b) were selected for metatranscriptomic analysis according to their different growth trajectories in a highly controlled flow-through system (Perera et al., 2019): fast-growth (family e6e2) and slow-growth (family c4c3). These animals, fed a control (D1) or a well-balanced plant-based diet (D2) for 9 months, were kept in eight 3,000 L tanks under a common garden system to eliminate confounding environmental effects. The total RNA from the intestinal mucus of the anterior portion of the gut was extracted from eight fish of each family and diet. Then, a total of 16 pooled samples (two fish of the same diet, family, and tank, replicate per sample) were sequenced, at the rate of four samples per experimental group. More details on the fish rearing and sampling can be found in Figure 1 and the Materials and Methods section.

### Sequencing and Metatranscriptome Assembly

Ribo-depletion and subsequent Illumina paired-end (PE) sequencing of the 16 pooled RNA samples yielded a total of 766 M reads (∼48 M reads per sample) (Supplementary Table 1). After trimming, quality filtering, and a second *in silico* ribosomal RNA removal step, around 3% of all reads were discarded, and the remaining reads ranged between 25 M (7.5 Gb) and 166 M (49.8 Gb) within the experimental groups. Pre-processed reads were then assembled and 358,784 unigenes (i.e., non-redundant transcripts) were identified (Table 1). Mapping of the cleaned reads (∼75%) resulted in all the unigenes being overlapped by, at least, one sequence.

**TABLE 1:** Metrics of the metatranscriptome reconstruction.

	Metatranscriptome assembly metrics
Assembly length (Mb)	250.26
Number of Unigenes	358,784
Min_length (bp)	251
Mean_length (bp)	698
Max_length (bp)	50,877
Annotated unigenes*	35,144
Unique descriptions	17,618
Hypothetical proteins	1,813
Uncharacterized and unnamed proteins	267

*Matched with Bacteria, Fungi, Archaea, and Virus sequences extracted from the NCBI’s NR database.

The unigenes alignment with bacterial, fungal, archaeal, and viral sequences extracted from the NCBI’s NR database to obtain the repertoire of genes expressed by microbial communities in the gut of gilthead sea bream, resulted in a total of 35,144 transcripts, which corresponded to ∼10% of the total assembled RNA transcripts (Table 1). These transcripts corresponded to 17,618 unique descriptions with a low proportion of hypothetical (1,813; 5.2%) and uncharacterised/unnamed (267; 0.8%) proteins.

### Taxonomic Composition of Gilthead Sea Bream Metatranscriptome

**FIGURE 2:**
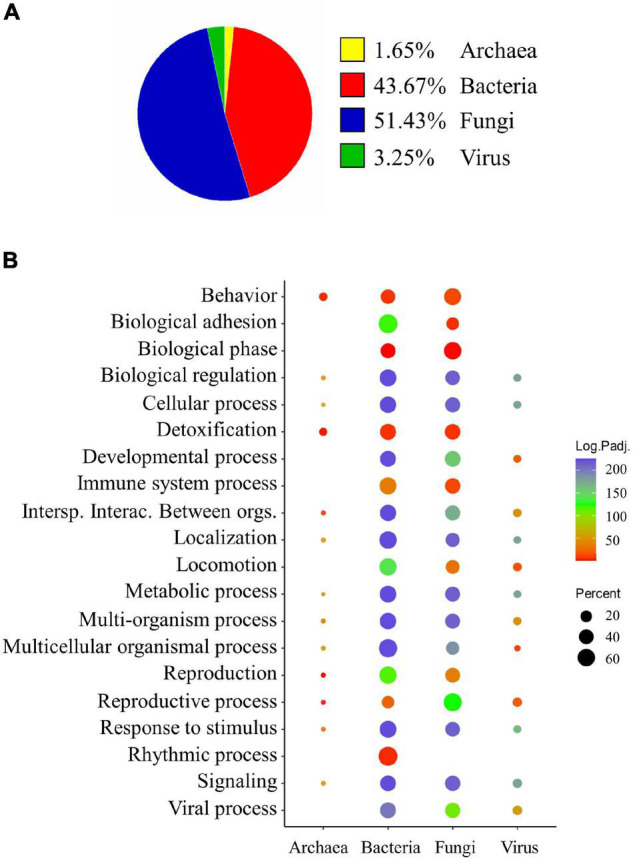
**(A)** Pie chart showing the relative expression of assembled unigenes associated to gut microbial communities according to their assigned taxonomy. **(B)** Dot plot depicting the results of an over-representation test performed over the Biological Process Gene Ontology terms (GO-BP) of each one of the assigned taxonomies. The size of the dots represents the percentage of genes in this category, which were retrieved with our assembly strategy in each group. The colour scale represents the –log_10_*Padj* value obtained in the over-representation test of each pathway within each taxonomy.

All 35,144 annotated unigenes of gilthead sea bream metatranscriptome were classified, at least, to one of the four targetted taxonomic kingdoms: bacteria, archaea, eukarya (for Fungi-related unigenes), and virus. Considering this assignation and the normalised gene expression level of the annotated unigenes, the relative expression of each taxonomy in all the samples was calculated. The results of this procedure showed that Fungi and Bacteria were the most active populations in our samples, representing 51.43 and 43.67% of the total gut microbial expression in our species, respectively. At a lower extent, genes belonging to Virus (3.25%) and Archaea (1.65%) populations were also expressed (Figure 2A).

The functional annotation of the taxonomic groups resulted in a diverse set of functional biological process categories (GO-BP) allocated to 23,706 annotated unigenes (11,284 unique descriptions). Then, an over-representation analysis inferred the terms associated with the intestinal microbial taxonomies, and 437 GO-BP were considered significantly over-represented (Fisher test; FDR < 0.05) among the different groups. To have an overview of the functionality of each microbial population in the gut, these over-represented terms were clustered in 20 level 2 GO-BP categories, present, at least, in one of the groups (Figure 2B). The genes expressed by the predominant bacterial and fungal populations disclose involvement in practically all the processes. However, the rhythmic process was concomitant with bacteria, whereas the immune system process was exclusively associated with bacterial and fungal communities. In addition, all taxonomic groups had an over-representation of routes related to interspecies interaction between organisms, multi-organism process, response to stimulus, and signalling terms, among others.

At the phylum level, an important proportion (68.22%) of the mapped reads assigned to Archaea was also assigned to Euryarchaeota (Supplementary Figure 1A). Otherwise, only 16.3, 6.03, and 30.92% of the Bacteria-, Fungi-, and Virus-assigned unigenes, respectively, were classified up to this taxonomic level (Supplementary Figures 1B–D). Proteobacteria (12.45% of the mapped reads), Bacteroidetes (2.44%), Firmicutes (0.56%), and Actinobacteria (0.43%) phyla were the most expressed among phylum-assigned bacterial transcripts. Ascomycota (1.85%) and Mucoromycota (1.45%) were the predominant phyla among fungi transcripts, whereas Herpesviridae (11.14%) and Retroviridae (10.65%) were the most metabolically active families among the unigene fraction of virus.

### Diet and Family Effects on Metatranscriptome Composition

To test if the genetic background or the diet influenced the expression of transcripts of the different microbial communities, changes in their relative expression abundances were assessed. Not considering the diet, the family variable showed no statistically significant effect on the metatranscriptome composition, as well as the interaction between family and diet variables. However, statistical differences (Two-way ANOVA, *P* < 0.05) were detected when the variable diet was studied independently (Table 2). Specifically, a trade-off between the relative abundance of bacteria (decreased from 45.18% in fish-fed D1 to 42.16% in fish-fed D2) and fungal (increased from 50.1% in fish fed D1 to 52.76% in fish fed D2) transcripts were found.

**TABLE 2:** Effects of genetic background and diet in the gilthead sea bream metatranscriptome composition.

	Group				Two-way ANOVA		
	c4c3-D1	c4c3-D2	e6e2-D1	e6e2-D2	Diet	Family	Interaction
Archaea	1.65 ± 0.11	1.83 ± 0.09	1.49 ± 0.04	1.61 ± 0.12	0.136	0.069	0.777
Bacteria	44.95 ± 0.88	42.16 ± 1.06	45.42 ± 0.54	42.17 ± 1.27	0.009*	0.810	0.815
Fungi	50.29 ± 0.08	52.81 ± 0.27	49.91 ± 0.04	52.72 ± 0.02	0.015*	0.808	0.876
Virus	3.11 ± 0.11	3.20 ± 0.27	3.18 ± 0.04	3.50 ± 0.03	0.063	0.088	0.290

Values are the mean ± SEM of four pooled samples (eight fish in total) per group. The P-values are the result of two-way analysis of variance. Bold numbers with an asterisk (*) represent significant P values (< 0.05).

**FIGURE 3:**
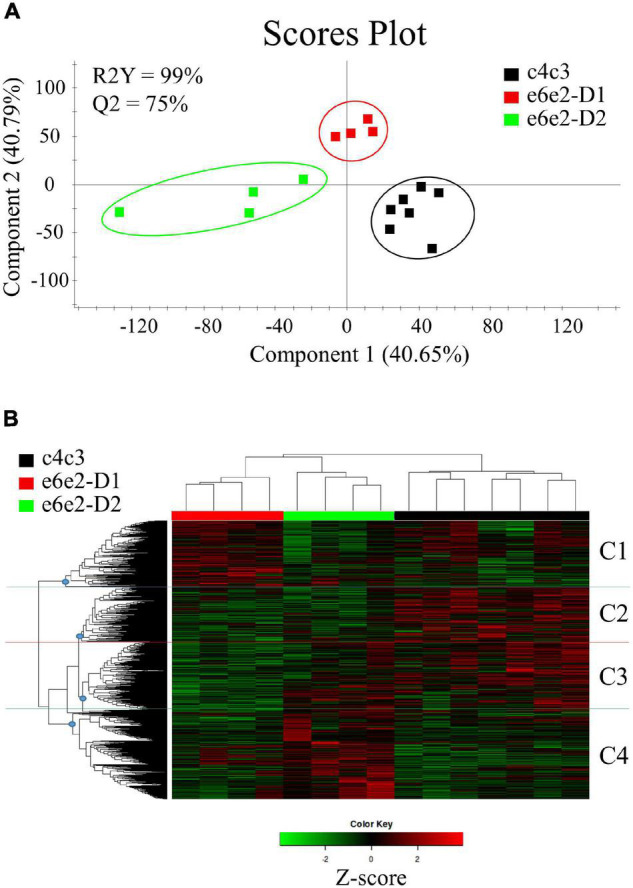
**(A)** Two-dimensional partial least-squares discriminant analysis (PLS-DA) score plot constructed using the FPKM values of assembled and annotated unigenes representing the distribution of the experimental groups between the first two components in the model. The validation by permutation test can be found in Supplementary Figure 2A. **(B)** Heatmap representing the abundance distribution (Z-score) of the genes identified to be driving the separation by diet (VIP ≥ 1). Letters next to the heatmap correspond to the four categories in which the clustering could be divided (dividing nodes are depicted as blue circles): C1: Up-regulated genes in e6e2-D1 with lower expression values in e6e2-D2 and c4c3; C2: Down-regulated genes in e6e2 fish in comparison to c4c3 fish; C3: Down-regulated genes in e6e2-D1 with higher expression values in e6e2-D2 and c4c3; C4: Genes up-regulated by the alternative diet (D2) in e6e2 family.

To further evaluate these differences in the microbial expression among the groups, a partial least squares discriminant analysis (PLS-DA), comprising the 35,144 annotated unigenes, was performed. The discriminant model was based on five components, which explained 99% [R2Y(cum)] and predicted 75% [Q2Y(cum)] of the total variance (Figure 3A). During the statistical processing to construct the model, one fish from the c4c3-D1 group, which coincided with the sample with the lowest number of sequenced reads, appeared as an outlier (Hotelling’s T^2^ > 0.99) and was excluded from the model. The fit of the resulting PLS-DA model was validated by a 500-random permutation test (Supplementary Figure 2A). The final model separated the c4c3 family from the e6e2 fish in the first component (∼41% explained variance), whereas the second component mainly separated the e6e2-D1 fish from the other two groups (∼41% explained variance). These results showed how D2 was significantly changing the metatranscriptomic profile in fast-growth families, but no differences were detected in slow-growth families when alternative diets were used. Similar results were found when different PLS-DA models were inferred using the annotated unigenes exclusively assigned to Bacteria (Supplementary Figures 2B,C), Fungi (Supplementary Figures 2D,E), Archaea (Supplementary Figures 2F,G), and Virus (Supplementary Figures 2H,I) groups. Likewise, a subsequent hierarchical clustering, using the FPKM expression values of the 5,998 genes driving the separation among groups (VIP ≥ 1), was not able to separate the samples from the c4c3 family fed both diets whereas the fish from e6e2-D1 and e6e2-D2 groups were assigned to different clusters (Figure 3B). Cluster analysis using the 5,998 differentially expressed genes, identified four gene clusters according to the expression levels in the different groups (optimal Elbow number = 4; Supplementary Figure 3): C1, 1,301 genes up-regulated in e6e2-D1 with lower expression values in e6e2-D2 and c4c3; C2, 1,007 genes down-regulated in e6e2 fish in comparison to c4c3 fish; C3, 1,502 genes down-regulated in e6e2-D1 with higher expression values in e6e2-D2 and c4c3; and C4, 2,188 genes up-regulated by the alternative diet (D2) in e6e2 family. Genes allocated to each group were used for further over-representation analysis. The functional annotation for the 5,998 genes overcoming the VIP threshold can be accessed in Supplementary Table 2.

### Functional Gene Ontology and Kyoto Encyclopaedia of Genes and Genomes Pathways Over-Representation Tests

To study the functionality of the genes involved in each clustered group, an over-representation test (Fisher test, FDR < 0.05) was performed. This procedure displayed 340 GO-BP and 236 Kyoto encyclopaedia of genes and genomes (KEGG) unique terms which were over-represented in, at least, one of the groups (Supplementary Figure 4A). Also, Venn diagrams showed a high degree of overlapping of terms between categories (Supplementary Figures 4B,C). To filter the list of over-represented terms avoiding intersections, only those unique terms for each group were retrieved and used for further analysis, with a total of 99 and 90 category-specific GO-BP and KEGG terms, respectively. The highest number of specific enriched GO-BP terms was found in C4 (54), followed by C3 (29), C1 (12), and C2 (4). In the case of enriched KEGG terms, 33 routes were found in C1, followed by C4 (25), C2 (18), and C3 (14). The entire list of over-represented terms in each group, and their respective list of associated genes can be accessed in Supplementary Tables 3, 4.

**FIGURE 4:**
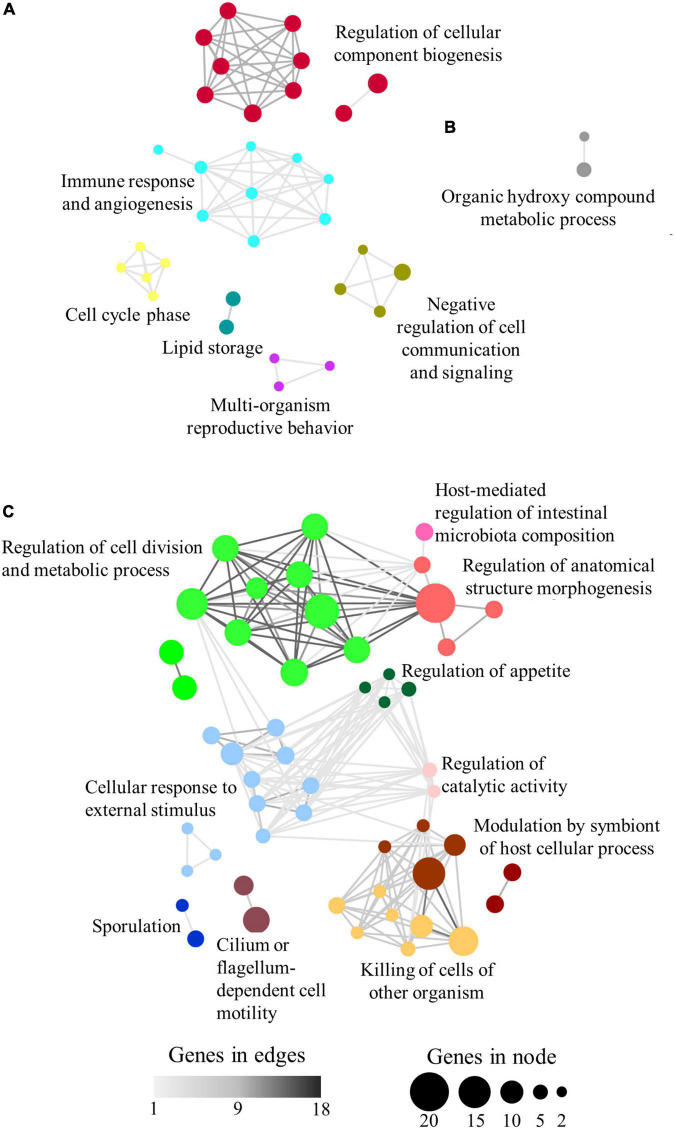
Network layout representing the associations between over-represented GO-BP terms according to their shared allocated genes in panels **(A)** C1 + C3, **(B)** C2, and **(C)** C4. Node size represents the number of genes allocated to a specific GO-BP term. Node colours show the representative name of clustered GO-BP terms. Edge width represents the number of shared genes between two GO-BP terms.

A total of 88 (89%) category-specific enriched GO-BP terms were found to be grouped according to their allocated shared genes. Thus, the over-represented terms associated with C1 and C3 groups were clustered in 6 supra-categories (Figure 4A). C1 and C3 were explored together as they present the same trend, similar expression in e6e2-D2 and c4c3, with significant differences only in e6e2-D1. The highest number of genes was present in the supra-category Regulation of cellular component biogenesis (20 microbial genes allocated to 10 GO-BP over-represented terms), followed by Negative regulation of cell communication and signalling (6 genes to 4 GO-BP), Immune response and angiogenesis (5 genes to 9 GO-BP), Lipid storage (5 genes to 2 GO-BP), Cell cycle phase (2 genes to 5 GO-BP), and Multi-organism reproductive behaviour (2 genes to 3 GO-BP). Only two GO-BP terms were connected in C2, under a supra-category named Organic hydroxyl compound metabolic process, which encompassed five microbial genes (Figure 4B). In the case of C4, a total of 10 supra-categories were found. Interestingly, two of them were closely related to symbiotic processes: Modulation by symbiont of host cellular process (28 genes to 6 GO-BP), and Host-mediated regulation of intestinal microbiota composition (7 genes to 1 GO-BP) (Figure 4C). The rest of the C4 supra-categories were named Cellular response to external stimulus (35 genes to 11 GO-BP), Regulation of cell division and metabolic process (34 genes to 12 GO-BP), Regulation of anatomical structure morphogenesis (24 genes to 4 GO-BP), Killing of cells of another organism (17 genes to 7 GO-BP), Cilium or flagellum-dependent cell motility (16 genes to 2 GO-BP), Sporulation (9 genes to 2 GO-BP), Regulation of appetite (5 genes to 4 GO-BP), and Regulation of catalytic activity (5 genes to 2 GO-BP).

**FIGURE 5:**
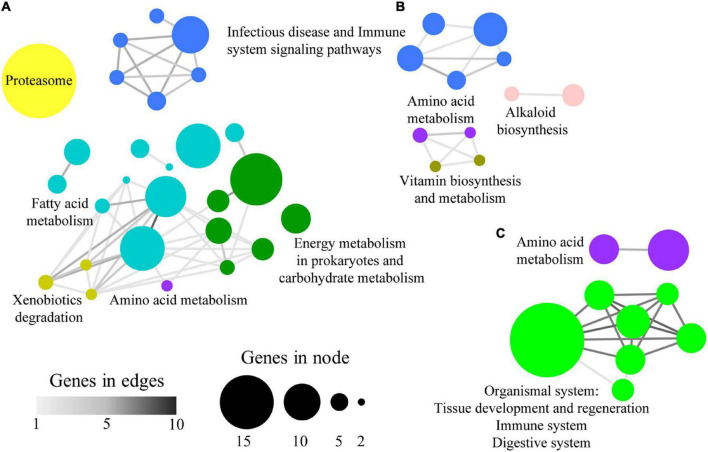
Network layout representing the associations between over-represented KEGG terms according to their shared allocated genes in panels **(A)** C1 + C3, **(B)** C2, and **(C)** C4. Node size represents the number of genes allocated to a specific GO-BP term. Node colours show the representative name of clustered KEGG terms. Edge width represents the number of shared genes between two KEGG terms.

The list of category-specific enriched KEGG terms was also clustered and nine supra-categories, containing 47 pathways (∼51%), were found. The groups C1 and C3 exclusively comprised the supra-categories Fatty acid metabolism (46 microbial genes allocated to 10 KEGG enriched terms), Energy metabolism in prokaryotes and carbohydrate metabolism (35 genes to 6 KEGG), Proteasome (34 genes to 1 KEGG), and Xenobiotics degradation (5 genes to 3 KEGG) (Figure 5A). Alkaloid biosynthesis (9 genes to 2 KEGG) and Vitamin biosynthesis and metabolism (4 genes to 2 KEGG) were limited to C2, whereas the Organismal system (26 genes to 7 KEGG) supra-category was restricted to C4 (Figures 5B,C). Terms related with Infectious diseases and Immune system signalling pathways (33 genes to 11 KEGG) were shared between C1–C3 and C2 group and the Amino acid metabolism’s (22 genes to 5 KEGG) supra-category was present in all groups.

### Taxonomic Composition of Enriched Supra-Categories

**FIGURE 6:**
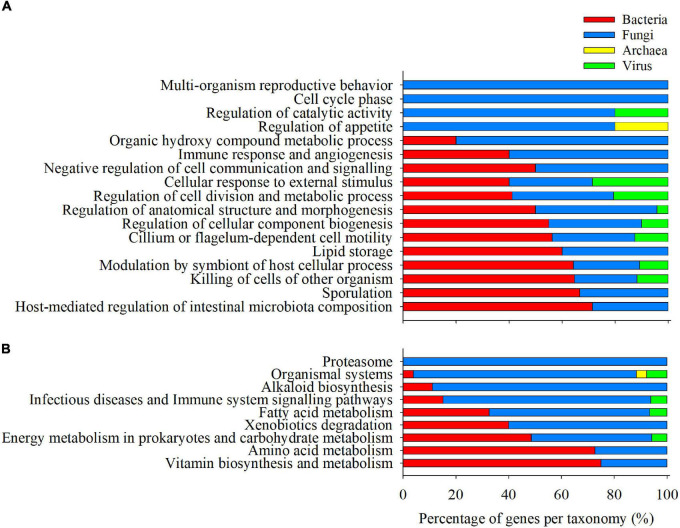
Barplot representing the relative abundance of Bacteria (red), Fungi (blue), Archaea (yellow), and Virus (green) transcripts within the fraction of genes allocated to each enriched and clustered GO-BP **(A)** and KEGG **(B)** category.

The list of category-specific, clustered, and enriched GO-BP (88) and KEGG (47) terms were allocated to 167 and 200 microbial transcripts, respectively. Among the GO-BP terms, a total of 73 genes (43.7%) were assigned to Bacteria, followed by Fungi (69; 41.3%), viruses (24; 14.4%), and Archaea (1; 0.6%). Fungal genes were predominant (>60%) in the supra-categories of Organic hydroxyl compound metabolic process, Regulation of catalytic activity, Regulation of appetite, and Immune response and angiogenesis (Figure 6A), whereas bacterial genes were more evident in the supra-categories of Modulation by symbiont of host cellular process, Sporulation, Host-mediated regulation of intestinal microbiota composition, Killing of cells of another organisms, and Lipid storage. On the other hand, a considerable amount of the genes allocated to enriched and clustered KEGG categories (140; 70%) were assigned to fungi, followed by bacteria (52; 26%), viruses (7; 3.5%), and Archaea (1;0.5%). The supra-category Proteasome and the categories associated with organismal systems (>60% of genes) were predominantly composed of fungal genes, as well as Alkaloid biosynthesis, Infectious diseases and immune system signalling pathways (Figure 6B). On the contrary, the Amino acid and Vitamin biosynthesis and metabolism supra-categories seem to be mainly directed by bacterial genes.

## Discussion

The gut microbiomes of fish are complex networks of communities, including members of bacteria, archaea, fungi, and viruses (Merrifield and Rodiles, 2015). However, it is often estimated that the abundance of bacterial taxa (>99%) outnumbers the proportion of the other microbial populations (<1%) (Merrifield and Rodiles, 2015; Egerton et al., 2018). In this scenario, amplicon-based sequencing techniques targetting the 16S rRNA bacterial gene have been helpful approaches, widely used to measure the composition and alterations of fish gut bacterial communities. However, these techniques do not offer the complete intestinal metagenome landscape, and microbial gene repertoire and expression cannot be retrieved or measured (Lindahl et al., 2013; Rausch et al., 2019). To overcome these limitations, this study reports a metatranscriptomic approach, showing that genes belonging to the Archaea, Bacteria, Eukarya (Fungi), and Virus domains are metabolically active in the gilthead seabream (Figure 2A). As expected, archaeal and viral transcripts were found in lower proportions (1.65 and 3.25%) in the intestinal mucosa, but bacterial and fungal transcripts were roughly equal (∼44 and ∼51%, respectively) and highly predominant in this mucosal surface. Indeed, several studies highlighted the importance of the highly diverse fungal microbiota fraction in humans (Huffnagle and Noverr, 2013; Santus et al., 2021). In the same line, more than 20% of the metatranscripts in the rumen of dairy cows have been related to fungi (Comtet-Marre et al., 2017), and untargeted metabolomics underlined the presence of fungal-derived metabolites in the serum of gilthead sea bream fed plant-based diets (Gil-Solsona et al., 2019). Certainly, the eukaryotic fungal cells, although lower in number, might become more transcriptionally active than prokaryotic cells.

As shown in Figure 2B, the functional annotation of the gilthead seabream metatranscriptome shared the over-representation of a significant number of GO-BP categories related to symbiosis (Interspecies interaction between organisms, Multi-organism process, Multicellular organismal process) and sensory responses (Behaviour, Response to stimulus, and Signalling), evidencing the contribution of all the microbial communities to the cooperative processes taking place in the host gut. The predominant taxa, Fungi and Bacteria, expressed genes related with all the GO-BP level 2 categories, but the Rhythmic process appeared as a bacterial-specific category. In this line, the manipulation of daily rhythms of gut bacterial microbiota abundance and activity is becoming a promising chrononutritional approach to consolidate host circadian rhythms and metabolic homeorhesis (Parkar et al., 2019; Gutierrez Lopez et al., 2021). Recently, Calduch-Giner et al. (2022) reviewed behavioural biosensing approaches based on accelerometer technology [AEFishBIT dataloggers (Rosell-Moll et al., 2021)] for informing on fish social behaviour in terms of coping styles or changes in daily or seasonal activity, linking ventilation rates with changes in energy partitioning between growth and physical activity. However, the association of changes in behaviour and gut microbiome rhythms remains almost unexplored in fish, and their inter-related study would contribute to discerning the disrupting effects of life stressors in the gut processes related to host rhythmicity. In any case, it is noticeable that the plant-enriched diet yielded a gilthead seabream metatranscriptome with a significant decrease of bacterial transcripts (∼7%), together with an increase (4%) in the number of fungal transcripts (Table 2) as described before in rainbow trout fed with yeast and soybean meal diets (Merrifield et al., 2009; Huyben et al., 2018).

In most cases, taxonomic assignment at lower taxonomic levels using the transcript sequences was not possible. Nonetheless, although commonly reported taxonomic assignments in amplicon-based protocols, such as families or genus, could not be related to their corresponding gene repertoire, the information shown in Supplementary Figure 1 can provide a hint of the most metabolically active phyla. Archaeal transcripts were majorly assigned to the Euryarchaeota phylum (Supplementary Figure 1A), one of the most discussed in humans for positively impacting gut health (Horz and Conrads, 2010). In line with their highest abundance in bacterial gut microbiome studies using 16S rRNA (Piazzon et al., 2017, 2019; Naya-Català et al., 2021b; Solé-Jiménez et al., 2021), the phyla Proteobacteria, Firmicutes, Actinobacteria, and Bacteroidetes were also the ones contributing with the most expressed bacterial transcripts when the axonomic assignment was possible (Supplementary Figure 1B). Ascomycota and Basidiomycota play a pivotal role in the expression of enzymes related to fish nutrition and intestinal maturation (Gatesoupe, 2007; Banerjee and Ghosh, 2014; Siriyappagouder et al., 2018), were the most abundant phyla among the fungal fraction (Supplementary Figure 1C). By last, within the viral fraction, *Herpesviridiae*, *Poxviridiae*, and *Retroviridiae* families appeared to be the transcriptionally prevailing and functional (Supplementary Figure 1D), as previously detected using wild and farmed gilthead sea bream (Filipa-Silva et al., 2020).

From a recent study (Piazzon et al., 2020), we concluded that gilthead sea bream families selected for fast-growth harboured a plastic bacterial microbiota that was able to adapt to diet changes with no impact on growth or health. Indeed, small changes in bacterial composition accounted for larger changes in metabolic capacity when the inferred metagenome and pathway analysis were conducted (59 metabolic pathways changing). Conversely, significant changes in intestinal bacterial composition were limited to changes in 15 metabolic pathways in fish families selected for slow-growth, assuming that all bacteria detected are metabolically active and expressing all their genes at a fixed level (Langille et al., 2013; Aßhauer et al., 2015; Louca et al., 2018). In line with these results, discriminant analysis of the metatranscriptome only showed a clear discriminant value with dietary changes for the fast-growth family (Figure 3A and Supplementary Figure 2). To measure the consistency of these results, the 5,998 discriminant genes were clustered in four groups (C1, C2, C3, and C4) (Figure 3B and Supplementary Figure 4), according to their expression pattern (discussed below). Clusters C1, C3, and C4 presented a differing expression pattern between the 6e2-D1 and e6e2-D2 groups. Over-represented KEGG terms in these clusters (Supplementary Table 4) disclosed that ∼70% (42 out of 59) of significant differentially expressed pathways predicted by the inferred metagenome of our previous study (Piazzon et al., 2020) were also detected in the current study, but a total of 218 unique enriched pathways were found. Altogether, although these results support that metagenome prediction tools can help to have an overview of the direction and magnitude of the metabolic changes, but metatranscriptomic analyses provide more complete and precise information.

The groups C1 and C3 encompassed 2,803 microbial genes whose expression pattern was influenced by the genetic selection for high growth and shifted toward values closer to slow-growth families with the plant-based diet (Figure 3B). Among the elements governing this difference in gilthead sea bream gut, we mainly found genes related to the principal metabolic routes, required in all microbial populations (Qin et al., 2010) (Figures 4A, 5A). Bacterial, fungal, and viral genes were associated with Fatty acid metabolism, the predominant supra-category in these groups. This is not surprising, as gut microbes can process lipid dietary components and perform processes not exerted by the host (Schoeler and Caesar, 2019). Within this supra-category, the predominant KEGG term was *N*-Glycan biosynthesis, exclusively exerted, according to our results, by fungal genes. Besides, genetic selection for growth was accompanied by a raise in the formation of these compounds, which are indigestible for the host and can be transformed into short-chain fatty acids by microbial fermentation in the gut lumen (Koropatkin et al., 2012). This pathway is enriched in C1, which suggests that the plant-based diet was down-regulating this pathway as part of a healthy and complex gut homeostatic process, although it is well-known that dietary butyrate supplementation can mitigate most of the inflammatory drawback effects of plant-based diets in gilthead sea bream (Estensoro et al., 2016; Piazzon et al., 2017). Together with the fatty acid metabolism, a wide representation of carbohydrate metabolism was found related to Bacteria and Fungi. Inside this supra-category, the pathway Glutathione metabolism was remarkably above the others. Glutathione is an anti-oxidative compound, widely distributed in the gastrointestinal tract of humans and rodents (Mardinoglu et al., 2015), acting as a growth and gut health health-promoting in trout (Wang et al., 2021). Here, the genetic selection for fast-growth induced an over-expression of microbial genes related to glutathione metabolism. However, this expression was reverted to values found in the slow-growing fish with the use of the plant-based diet. The bacterial fraction of the intestinal microbiota was predominantly expressing genes related to the Amino acid metabolism process in the C1 group, but also in C2 and C4. The difference among groups resided in the type of amino acid that this community is making available in the fish, being the control of dietary protein source a strategic approach for the control of amino acid-fermenting bacterial species and their metabolic pathways, which in turn could have an impact on the metabolism of the host gut (Neis et al., 2015). By last, in C3 and C2, we also found a strong association between infectious diseases and immune-inflammatory pathways, mediated by fungal genes. This is a usual link in the gut microbiomes (Lokesh et al., 2012; Al Bander et al., 2020) and yeasts have a protective role against fish pathogens by expressing immunostimulatory substances (Li and Gatlin, 2006; Lokesh et al., 2012). According to our results, the formation of these compounds would be regulated by both the diet and genetic background.

Group C2, comprising 1,007 genes, differentiated the genetic background of the samples with no diet effect, demonstrating the effect of selective breeding on active gut microbial populations (Figures 4B, 5B). In addition to the amino acid metabolism and the infectious disease and immune system supra-categories, formerly described, we found the fungal-associated Alkaloid biosynthesis supra-category. Traditionally, these bioactive compounds have been related to plants (Peng et al., 2019), but fungi, especially the Ascomycota phylum, are also able to produce them (Xu et al., 2020). The properties of these organic molecules include anti-microbial and anti-oxidant activity (Iranshahy et al., 2014), and their inclusion into diets have been suggested as possible alternatives to antimicrobial growth enhancers (Willems et al., 2020). In fish, these chemicals have been stated to produce anti-nutritional effects due to palatability issues when they were included in the diet of rainbow trout (Glencross et al., 2006). However, the positive properties of alkaloids cannot be underestimated, and the gut fungal population could be an interesting target at the time of exploring the microbial production of alkaloids for reducing the use of antibiotics in aquaculture production (Okocha et al., 2018).

Finally, a total of 2,188 genes were assigned to C4, a group showing the genetic × diet interaction effect in the fast-growth family. The expression of genes in this group remained attenuated in the fast-growing fish fed the control diet and was upregulated in fast-growth fish fed the plant-based diet, disclosing the dual response of this fish group when dealing with alternative diets (Figures 4C, 5C). Alternative diet formulations in gilthead sea bream are prone to produce changes in the intestinal plasticity of this species (Perera et al., 2019; Naya-Català et al., 2021b; Solé-Jiménez et al., 2021). Indeed, the families selected for growth used in this study showed an increased intestinal length when fed plant-enriched diets (Perera et al., 2019). Herein, the over-representation test disclosed the link between two supra-categories related to this anatomical feature: Host-mediated regulation of intestinal microbiota composition and Regulation of anatomical structure morphogenesis. These categories, mainly composed of bacterial transcripts, highlight an important role of this population in the intestinal reshaping upon feeding plant-enriched diets to fish families selected for fast growth. The bacterial population was also expressing genes related to the coupled categories Symbiont modulation of host cellular process and Killing of cells of other organisms. A recent study stated that diet and gut microbes could jointly act as enhancers of the programmed cell death to reduce colorectal cancer (Chapkin et al., 2020), and in humans, the bacterial community is a rich source of metabolites against pathogenic fungi, via the activation of the mTOR signalling pathway (Li et al., 2019b). Herein, this association suggests the role of bacterial intestinal symbionts in the modulation of processes resulting in the death or programmed death of host or other symbiont cells. Fungal genes in C4 were involved in the regulation of appetite and several processes related to Organismal systems interaction. However, the main expressed genes inside this supra-category were kinases and mitogen-activated protein kinases families, widely analyzed in fungi (Martínez-Soto and Ruiz-Herrera, 2017), which networked together with functions of tissue development and regeneration (Dorso-ventral formation) and digestive (Bile secretion), endocrine (Aldosterone and Prolactin signalling pathways), and immune (Chemokine signalling pathway) functions. It is widely documented that gut microbial metabolites can play a pivotal role in the regulation of these functions (Tan et al., 2018; Silva et al., 2020). However, the current results just suggest that the introduction of plant-enriched diets to fast-growth families is changing the signal transduction processes in the fungal symbionts, and only further metabolomics studies could help to discern the resulting metabolites of these signalling cascades.

To sum up, this metatranscriptomic approach was very useful for measuring which microbial populations are metabolically active in the anterior intestine of gilthead sea bream and revealed a wide range of processes carried out by microbes that can serve as a gene catalogue for future studies. Moreover, all the transcripts were taxonomically assigned to the level of the kingdom, so processes exerted predominantly by a specific gut community could be disclosed at this level. In this line, 18S rRNA amplification approaches measuring the composition and variations of fungal intestinal communities arise as promising targets to completely understand the processes occurring in the anterior part of this tissue in gilthead sea bream. Furthermore, despite the simplicity of the experimental model, where only two families selected for growth were used, this study helped us to corroborate the higher functional plasticity of the microbiome of fish selected for fast growth, which was able to shape a changing metatranscriptome with a more stable metagenome.

## Materials and Methods

### Ethics Statement

All procedures were approved by the Ethics and Animal Welfare Committee of IATS and CSIC. They were carried out in a registered installation facility (code ES120330001055) in accordance with the principles published in the European Animal Directive (2010/63/EU) and Spanish laws (Royal Decree RD53/2013) for the protection of animals used in scientific experiments.

### Experimental Setup and Sampling

The growth-selected gilthead sea bream families used in this study were obtained from the Spanish selection program of gilthead sea bream (PROGENSA^®^) and reared as previously described (Perera et al., 2019). Briefly, fish from families e6e2 (fast-growth) and c4c3 (slow-growth) were randomly distributed (common garden system) in eight 3,000 L tanks under a flow-through system and natural photoperiod and temperature at the IATS facilities (Castellón, Spain: 40° 5′N; 0° 10′E). Fish were individually tagged in the dorsal muscle with passive integrated transponders (PIT) and mixed in equal proportions and with a similar number of family members in each tank. During 9 months, four tanks were fed a control diet (D1) and the other four a well-balanced plant-based diet (D2). The exact composition of the diets and details on fish rearing can be found elsewhere (Perera et al., 2019; Supplementary Table 5).

At the end of the feeding trial (July 2018), a total of 32 (four fish, two per family, per tank) 48-h fasted fish (males) with a mean bodyweight of ∼138 g (D1), and ∼130 g (D2) were anaesthetized with 0.1 g/L of tricaine-methanesulfonate (MS-222, Sigma-Aldrich, St. Louis, MO, United States) and sacrificed by cervical section. The anterior intestine was, then, cut out, opened, and washed with phosphate-buffered saline (PBS) to remove non-adherent materials and microbes. The tissue was transferred to a clean Petri dish, and the intestinal mucus was scraped out with the blunt end of a sterile scalpel. The sampled mucus was immediately frozen in liquid nitrogen and kept at −80°C until microbial RNA extraction.

### RNA Extraction, Illumina Sequencing, and Sample Quality Assessment

For RNA extraction 200 μl of intestinal mucus were mixed with 500 μl of TriReagent (Invitrogen, Waltham, MA, United States) and microbes were lysed in microbial lysis tubes (Qiagen, Germantown, MD, United States) using 1 cycle of 30 s at 6 m/s in a FastPrep homogenizer (MP Biomedicals, Irvine, CA, United States). Total RNA was extracted using the MagMAX™-96 for Microarrays Total RNA isolation kit (Life Technologies, Carlsbad, CA, United States) following the manufacturer’s instructions. The quality and integrity of the isolated RNA were checked on an Agilent Bioanalyzer 2100 total RNA Nano series II chip (Agilent) with RIN (RNA Integrity Number) values varying between 8 and 10. For further procedures, a total of 16 pooled samples were used. Each pool contained an equimolar amount of RNA of two individuals of the same diet, family and tank. After quality and integrity procedures, rRNA was removed using the Illumina Ribo-Zero Plus rRNA Depletion Kit (Illumina Inc., San Diego, CA, United States), which targets both eukaryotic and prokaryotic rRNA. Then, Illumina RNA-seq libraries were prepared from 500 ng of total ribo-depleted RNA using the Illumina TruSeq*™* Stranded Total RNA Library Prep Kit (Illumina Inc., San Diego, CA, United States) according to the manufacturer’s instructions.

All RNA-seq libraries were sequenced on an Illumina NovaSeq 6000 platform as a 2 × 250 nucleotides paired-end (PE) read format, according to the manufacturer’s protocol. Raw sequenced data were deposited in the Sequence Read Archive (SRA) of the National Centre for Biotechnology Information (NCBI) under the Bioproject accession number PRJNA790012 (BioSample accession numbers: SAMN24182635-50). Quality analysis of sequencing reads was performed with FASTQC v0.11.7 (last accessed: 23 April 2020),^1^ and libraries were pre-processed with Trimmomatic v0.40 (Bolger et al., 2014), removing reads with adaptor contamination, >10% of Ns in the sequence, and with a mean sequence quality < 20. To ensure the elimination of rRNA sequences, filtered reads were aligned to rRNA and tRNA databases from NCBI (Altschul et al., 1990) and SILVA databases (Quast et al., 2013). The remaining sequences were used for further steps.

### Bioinformatics Analysis

Cleaned reads were introduced in Trinity v.2.11.0 (Grabherr et al., 2011) for the *de novo* transcriptome reconstruction, setting a k-mer length of 25 and minimum k-mer coverage of 2. Assembled transcripts were clustered at 95% identity threshold for redundancy removal using CD-HIT v.4.6 (Fu et al., 2012) to obtain unigenes. Alignments against the Bacteria, Fungi, Archaea, and Virus sequences extracted from the NCBI’s NR database were performed with DIAMOND v.0.8.22 (Buchfink et al., 2014) using the blastx algorithm option (e-value < 10^–5^). The same algorithm was used to compare the non-aligned fraction of transcripts against the gilthead sea bream genome (Pérez-Sánchez et al., 2019) to confirm host origin (e-value < 10^–5^). These host sequences were not used in downstream analyses. The Lowest Common Ancestor (LCA) algorithm, implemented in the MEGAN software (Huson et al., 2018), was used to taxonomically classify the microbial-aligned sequences without losing biological significance. Since multiple alignments may occur, this algorithm assigned each unigene to the lowest node in the NCBI taxonomy that encompasses the set of NR-aligned sequences, when possible. To calculate the gene expression levels, cleaned reads were mapped against the reconstructed unigene metatranscriptome as a reference using Bowtie2 v.2.4.4 (Langmead and Salzberg, 2012). Mapping results were analysed using RSEM v1.2.15 (Li and Dewey, 2011), which rendered the read count for each gene in each sample. Then, read counts were normalised into FPKM to consider the effects of both sequencing depth and gene length.

Functional annotation of GO-BP and KEGG metabolic pathways was performed over the assembled and annotated unigenes model using blast2go (e-value ≤ 10^–6^) and DIAMOND (Buchfink et al., 2014), respectively. The GO-BP terms’ hierarchy was retrieved using the QuickGO API tool (last accessed: August 2021)^2^ and GO-BP were clustered according to their ancestor in Gene Ontology (GO) at level 2 (i.e., immediate child of Biological process; GO:0008150). Fisher test-based over-representation tests of BP-GO and KEGG terms were implemented in the *goseq* R package (Young et al., 2010). In the case of enriched KEGG categories, once the over-representation test was performed, all enriched terms belonging to processes associated to human and non-related to microbial species were excluded. The relationships between enriched GO-BP and between KEGG terms according to their shared genes were performed using the *runGSA* function of *piano* R package (Väremo et al., 2013), and the resulting networks were visualised with Cytoscape v3.8.2 (Smoot et al., 2011).

### Statistics

Effects of genetic background and diet on the relative abundance of microbial transcripts expression were analyzed by two-way ANOVA using SigmaPlot v.14.5 (Systat Software Inc.). Data was previously checked for normal distribution (Shapiro–Wilk test) and homogeneity of variances (*F*-test). To study the separation among the groups, supervised PLS-DA and hierarchical clustering of samples were performed using EZinfo v3.0 (Umetrics) and R package ggplot2, respectively. Values of FPKM counts of genes expressed in five or more samples were included in the analyses. The contribution of the different genes to the group separation was determined by the minimum variable importance in the projection (VIP) values achieving the complete clustering of the conditions with a VIP value ≥ 1, considered to be an adequate threshold to determine discriminant variables in the PLS-DA model (Wold et al., 2001; Li et al., 2012; Kieffer et al., 2016). Hotelling’s T2 statistic (at 99% range) was calculated with the multivariate software package Ezinfo v3.0 to detect outliers in the model. The quality of the PLS-DA model was evaluated by the parameters R2Y (cum) and Q2 (cum), which indicate fit and prediction ability, respectively. To assess whether the supervised model was being over-fitted, a validation test consisting of 500 random permutations was performed using the Bioconductor R package *ropls* (Thévenot et al., 2015). The optimal number of categories in which the clustering of genes could be divided was determined through the Elbow method using the *stats* R package. For this purpose, the within-group sum of squares at each number of clusters (from 1 to 10) was calculated and graphed. The location of the bend in the plot was considered the appropriate number of nodes. Significantly enriched GO-BP and KEGG categories were obtained after FDR correction using a cut-off of 0.05.

## Data Availability Statement

Raw sequencing data can be found at NCBI’s Sequence Read Archive under accession PRJNA790012 (BioSample accession numbers: SAMN24182635-50).

## Ethics Statement

The animal study was reviewed and approved by Ethics and Animal Welfare Committee of IATS and CSIC.

## Author Contributions

AS-B and JP-S: conceptualisation, funding acquisition, project administration, resources, and supervision. FN-C, MCP, and JP-S: data curation, formal analysis, and writing—original draft. FN-C and MCP: visualisation. FN-C, MCP, JC-G, AS-B, and JP-S: investigation, writing—review and editing, and read and approved the final manuscript. All authors contributed to the article and approved the submitted version.

## Author Disclaimer

This publication reflects only the authors’ view and the European Union cannot be held responsible for any use that may be made of the information contained herein.

## Conflict of Interest

The authors declare that the research was conducted in the absence of any commercial or financial relationships that could be construed as a potential conflict of interest.

## Publisher’s Note

All claims expressed in this article are solely those of the authors and do not necessarily represent those of their affiliated organizations, or those of the publisher, the editors and the reviewers. Any product that may be evaluated in this article, or claim that may be made by its manufacturer, is not guaranteed or endorsed by the publisher.

## Footnotes

1
https://www.bioinformatics.babraham.ac.uk/projects/fastqc

2
https://www.ebi.ac.uk/QuickGO/api/index.html
