# Supplementary material for: Diet and Host Genetics Drive the Bacterial and Fungal Intestinal Metatranscriptome of Gilthead Sea Bream
Source: Front Microbiol. 2022 May 6;13:883738. doi: 10.3389/fmicb.2022.883738 (PMC9121002; doi:10.3389/fmicb.2022.883738)

Supplementary Figure 1. Pie charts showing the relative expression of assembled unigenes associated to gut microbial communities according to their phylum within (A) Archaeal, (B) Bacterial, (C) Fungal, and (D) Viral unigenes.

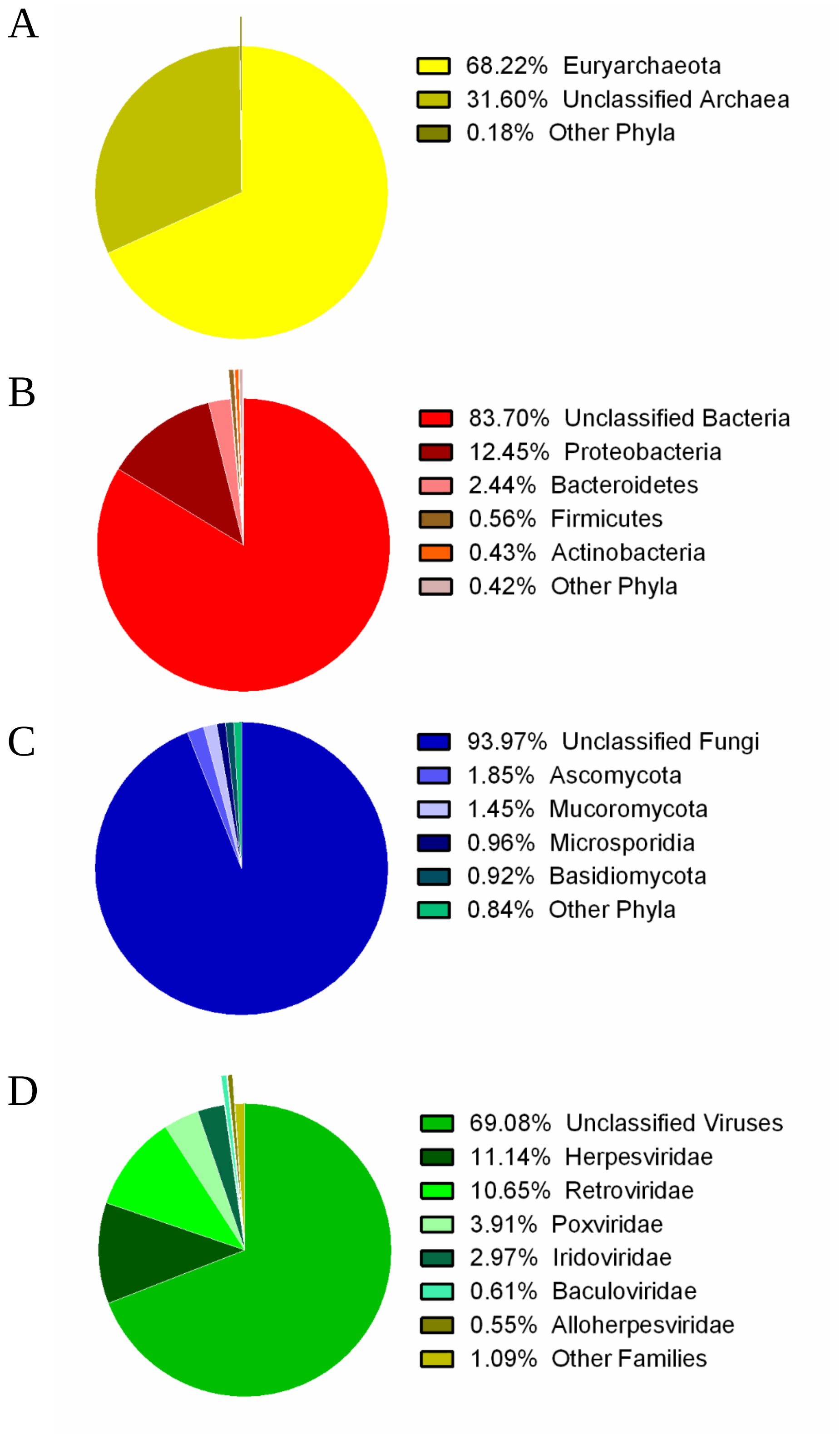

Supplement: Supplementary file 1 [file Data_Sheet_1.PDF]
