# Supplementary material for: Diet and Host Genetics Drive the Bacterial and Fungal Intestinal Metatranscriptome of Gilthead Sea Bream
Source: Front Microbiol. 2022 May 6;13:883738. doi: 10.3389/fmicb.2022.883738 (PMC9121002; doi:10.3389/fmicb.2022.883738)

Supplementary Figure 2. (A) 500-random permutations validation test of the PLS-DA model shown in Fig. 3A of this study. Two-dimensional partial least-squares discriminant analysis (PLS-DA) score plots, and their respective 500-random permutations validation plots, constructed using only the FPKM values of assembled and annotated unigenes assigned to (B, C) Bacteria, (D, E) Fungi, (F, G) Archaea, and (H, I) Virus taxonomies.

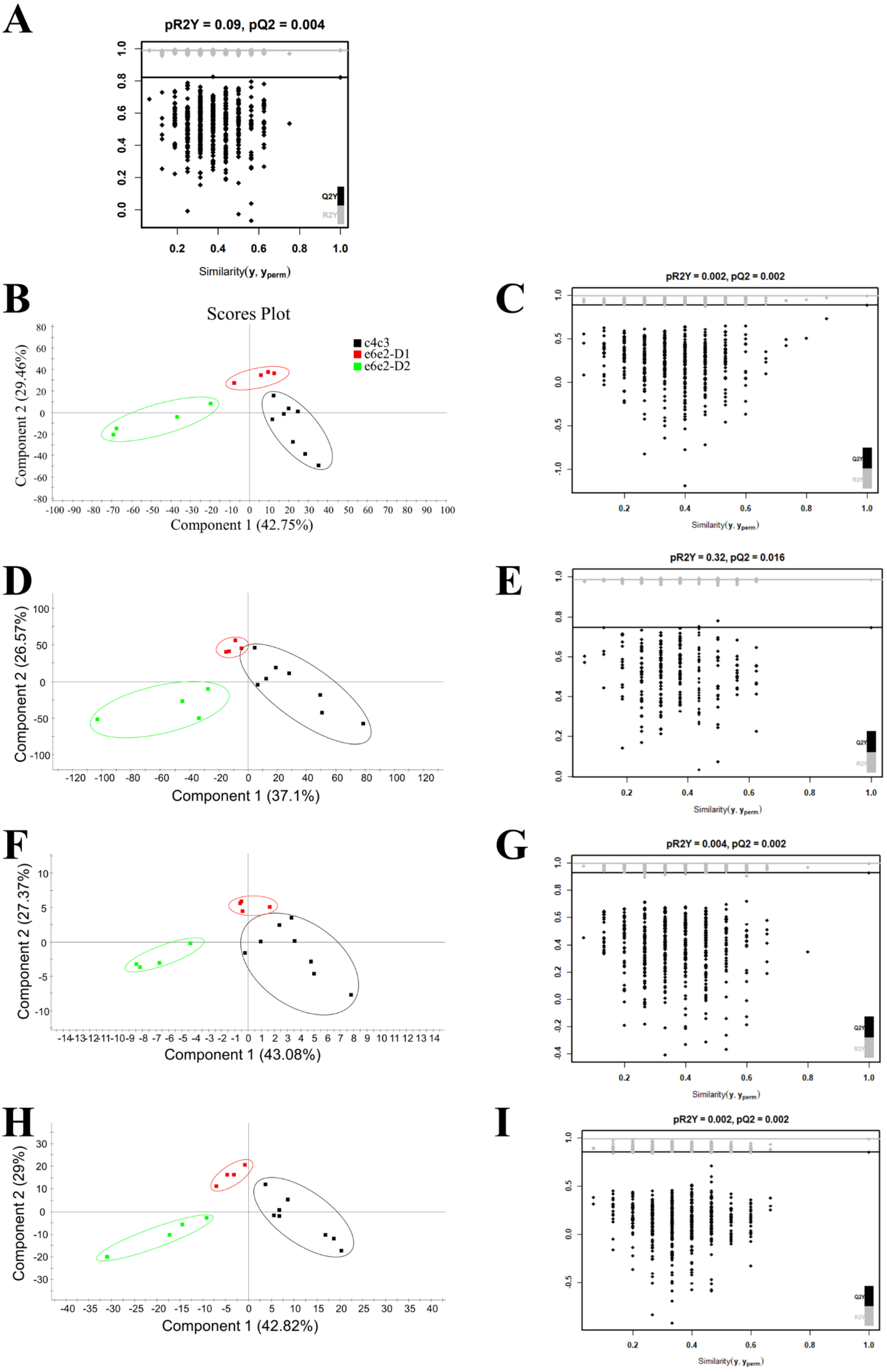

Supplement: Supplementary file 2 [file Data_Sheet_2.PDF]
