# Supplementary material for: Diet and Host Genetics Drive the Bacterial and Fungal Intestinal Metatranscriptome of Gilthead Sea Bream
Source: Front Microbiol. 2022 May 6;13:883738. doi: 10.3389/fmicb.2022.883738 (PMC9121002; doi:10.3389/fmicb.2022.883738)

Supplementary Figure 3. Results of the Elbow method used to find the optimal number of clusters for the 5,998 genes with VIP ≥ 1. The within group sum of squares at each number of analysed clusters (from 1 to 10) was plotted. The dotted line location indicates the bend in the plot, which makes k = 4 the appropriate number of nodes.

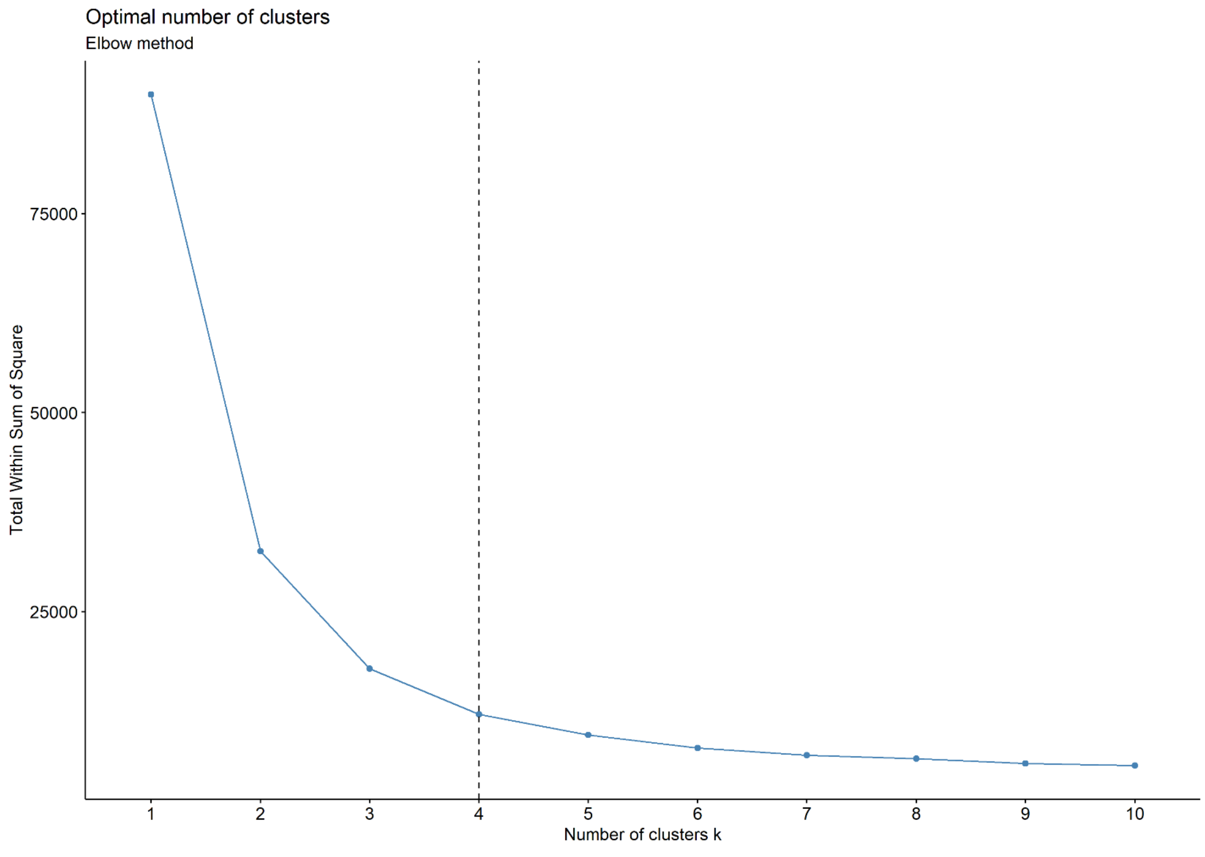

Supplement: Supplementary file 3 [file Data_Sheet_3.PDF]
