# Supplementary material for: Diet and Host Genetics Drive the Bacterial and Fungal Intestinal Metatranscriptome of Gilthead Sea Bream
Source: Front Microbiol. 2022 May 6;13:883738. doi: 10.3389/fmicb.2022.883738 (PMC9121002; doi:10.3389/fmicb.2022.883738)

Supplementary Figure 4. (A) Main results of the over-representation test in each one of the categories of the clustering. Venn diagrams showing the overlapping of over-represented (B) GO-BP and (C) KEGG terms within the categories of the clustering of discriminant microbial gene expression values.

A

	C1	C2	C3	C4	TOTAL
**Biological Significance**	**↑ in e6e2-D1; ↓ e6e2-D2 & c4c3**	**Genetics effect**	**↓ in e6e2-D1; ↑ e6e2-D2 & c4c3**	**Diet effect in fast-growing family**	
**#Genes**	1,301	1,007	1,502	2,188	**5,998**
**Enriched GO**	175	200	218	281	**340**
**Genes in Enriched GO**	821	676	1019	1547	**4,063**
**Enriched KEGG**	138	132	94	101	**236**
**Genes in Enriched KEGG**	411	251	235	256	**1,153**
**Non-overlapping GO**	12	4	29	54	**99**
**Non-overlapping KEGG**	33	18	14	25	**90**

B

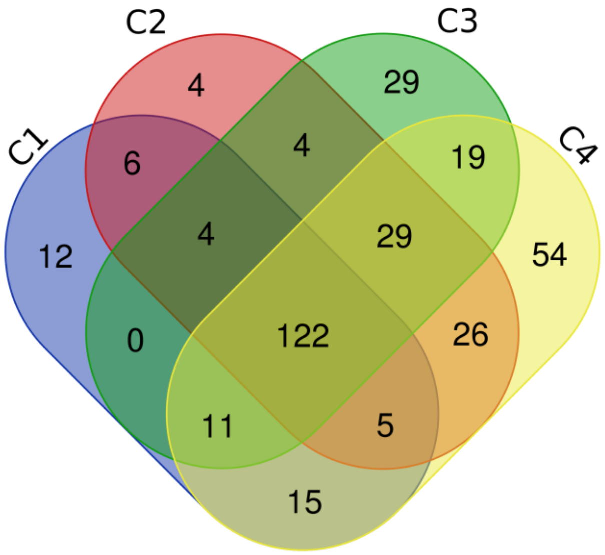

C

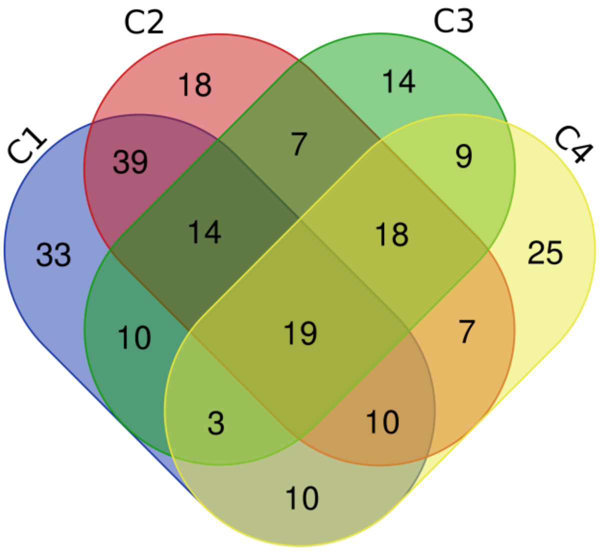

Supplement: Supplementary file 4 [file Data_Sheet_4.PDF]
