# Supplementary material for: Diet and Host Genetics Drive the Bacterial and Fungal Intestinal Metatranscriptome of Gilthead Sea Bream
Source: Front Microbiol. 2022 May 6;13:883738. doi: 10.3389/fmicb.2022.883738 (PMC9121002; doi:10.3389/fmicb.2022.883738)
Supplement: Supplementary file 5 [file Table_1.PDF]

Supplementary Table 1. Sequencing metrics for each sample of the metatranscriptomics study.

Family	Diet	Replicate	Raw reads	Clean reads	Percentage (%)	Mapped reads	Percentage (%)
c4c3	D1	1	40,891,462	39,503,218	96.61	29,535,236	74.77
c4c3	D1	2	44,733,288	43,475,676	97.19	33,411,698	76.85
c4c3	D1	3	41,611,062	40,378,238	97.04	29,525,320	73.12
c4c3	D1	4	25,899,406	25,114,062	96.97	17,957,140	71.5
c4c3	D2	1	41,431,038	40,553,498	97.88	31,664,118	78.08
c4c3	D2	2	42,755,210	41,751,314	97.65	31,957,790	76.54
c4c3	D2	3	38,247,064	37,281,302	97.47	27,184,404	72.92
c4c3	D2	4	37,874,000	36,937,354	97.53	28,017,292	75.85
e6e2	D1	1	40,122,332	38,935,624	97.04	29,737,172	76.38
e6e2	D1	2	40,807,312	39,231,852	96.14	30,455,580	77.63
e6e2	D1	3	42,462,638	41,424,984	97.56	30,882,288	74.55
e6e2	D1	4	38,302,598	36,969,424	96.52	28,004,748	75.75
e6e2	D2	1	43,645,580	41,931,378	96.07	32,076,840	76.5
e6e2	D2	2	169,671,998	166,559,008	98.17	120,864,246	72.57
e6e2	D2	3	37,134,958	36,248,832	97.61	26,471,636	73.03
e6e2	D2	4	40,909,058	39,588,022	96.77	29,290,554	73.99
