# Supplementary material for: Diet and Host Genetics Drive the Bacterial and Fungal Intestinal Metatranscriptome of Gilthead Sea Bream
Source: Front Microbiol. 2022 May 6;13:883738. doi: 10.3389/fmicb.2022.883738 (PMC9121002; doi:10.3389/fmicb.2022.883738)
Supplement: Supplementary file 9 [file Table_5.PDF]

Supplementary Table 5. Ingredients and chemical composition of experimental diets.

**Ingredient (%)**	**D1**	**D2**
Fish meal	23.0	3.0
Fish hydrolysate (CPSP)	2.0	2.0
Soya protein	16.7	25.6
Corn gluten	16.5	25.5
Wheat gluten	4.5	7.3
Rapeseed cake	12.0	10.0
Wheat	10.0	7.4
Fish oil	14.1	3.9
Rapeseed oil	0	9.0
Mineral-vitamin mix[a]	1.25	6.3
Proximate composition (%)		
Moisture	7.9	7.5
Crude protein	45.0	45.0
Crude fat	20.1	20.1
Ash	6.9	5.9
NFE[b]	19.1	19.8
ARA[c]	0.17	0.05
EPA[d]	2.30	0.60
DHA[e]	1.50	0.42
EPA + DHA	3.8	1.02
Crude energy (MJ/kg)	22.1	22.3

[a] Contains vitamins, minerals, amino acids, cholesterol, lecithin and anti-oxidants.
[b] Nitrogen-free extract.
[c] Arachidonic acid (20:4n-6).
[d] Eicosapentaenoic acid (20:5n-3).
[e] Docosahexaenoic acid (20:6n-3).
